# Draft Genome Sequence of *Cylindrospermopsis raciborskii* (Cyanobacteria) Strain ITEP-A1 Isolated from a Brazilian Semiarid Freshwater Body: Evidence of Saxitoxin and Cylindrospermopsin Synthetase Genes

**DOI:** 10.1128/genomeA.00228-16

**Published:** 2016-05-05

**Authors:** Adriana Sturion Lorenzi, Genivaldo Gueiros Z. Silva, Fabyano Alvares Cardoso Lopes, Mathias Ahii Chia, Robert A. Edwards, Maria Carmo Bittencourt-Oliveira

**Affiliations:** a Department of Biological Sciences, Laboratory of Cyanobacteria, Luiz de Queiroz College of Agriculture, University of São Paulo (USP), Piracicaba, São Paulo, Brazil; b Computational Science Research Center, San Diego State University, San Diego, California, USA; c Department of Cell Biology, Laboratory of Enzymology, University of Brasília (UNB), Brasília, Brazil; d School of Marine and Atmospheric Sciences, Stony Brook University, Southampton Campus, New York, USA; e Department of Computer Science, San Diego State University, San Diego, California, USA

## Abstract

*Cylindrospermopsis raciborskii* ITEP-A1 is a saxitoxin-producing cyanobacterium. We report the draft genome sequence of ITEP-A1, which comprised 195 contigs that were assembled with SPAdes and annotated with Rapid Annotation using Subsystem Technology. The identified genome sequence had 3,605,836 bp, 40.1% G+C, and predicted 3,553 coding sequences (including the synthetase genes).

## GENOME ANNOUNCEMENT

*Cylindrospermopsis raciborskii* (Woloszynska) Seenayya and Subba Raju populations of Brazilian freshwater bodies are potential saxitoxin (STX) producers (1), and the presence of the *sxt* biosynthetic gene cluster has been confirmed in the T3 strain (2). However, cylindrospermopsin (CYN), a cyanotoxin commonly detected in isolates from Australia and Asia, has recently been reported in Brazilian and South American water supply reservoirs containing *C. raciborskii* for the first time (3). Both cyanotoxins pose significant risk to human and animal health, because CYN causes general cytotoxic, hepatotoxic, and neurotoxic effects, and STX has been implicated in paralytic shellfish poisoning syndrome.

*C. raciborskii* ITEP-A1 was isolated from Arcoverde reservoir, Pernambuco, Brazil (8°33′32.5″S, 36°59′07.5″W). Genomic DNA was extracted from unicyanobacterial cells using the PowerSoil DNA isolation kit (MO BIO Laboratories, Inc., USA) and quantified using a Qubit Fluorometer (Life Technology, USA). Whole-genome sequencing was performed via the MiSeq personal sequencing system (Illumina, USA), using the 500-cycle MiSeq reagent kit version 2 (Illumina). The quality of paired-end reads was checked with FastQC version 0.11.3 (4). PRINSEQ version 0.20.4 (5) was used to trim quality scores under Phred 25, and sequences shorter than 200 bp were removed. Cyanobacterial sequence reads were obtained using the CLARK full mode (confidence score ≥0.75 and gamma score ≥0.03) (6), and published genome sequences of the *Nostocaceae* family as references. *De novo* genome assembly was carried out with SPAdes version 3.1.1 (7), and annotation performed via the RAST server (8) for contigs equal or longer than 1,000 bp. The final draft genome assembly consisted of 195 contigs, 3,605,836 bp (G+C content of 40.1%), and an *N*_50_ value of 91,008. It comprises 3,553 coding sequences of genes, 43 predicted RNA genes (42 tRNA genes and 1 rRNA gene), and 352 subsystems, which represent 40% of assigned sequences. Genome analysis revealed for the first time that ITEP-A1 possesses fragments of gene cluster responsible for cylindrospermopsin biosynthesis, while the presence of saxitoxin genes was confirmed. Genes encoding heterocyst formation, nitrogen fixation, and stress response (oxidative stress, osmotic stress, and heat shock proteins) were found. Genomic analysis revealed that *C. raciborskii* is capable of evolving diverse genomic organization and adaptive mechanisms.

### Nucleotide sequence accession numbers.

The draft genome sequence of *C. raciborskii* ITEP-A1 has been deposited at DDBJ/ENA/GenBank under the accession number LUBZ00000000. The version described in this paper is the first version, LUBZ01000000.
